# Social network analysis of the biblical Moses

**DOI:** 10.1007/s41109-016-0012-1

**Published:** 2016-11-14

**Authors:** Steven E. Massey

**Affiliations:** Department of Biology, University of Puerto Rico – Rio Piedras, San Juan, PR 00931 USA

**Keywords:** Pentateuch, Moses, Social network, Assortativity, Emergent, Power law with exponential cutoff

## Abstract

Here, social network analysis approaches are used to characterize the figure of the biblical Moses, and his relationship with characters from the books of the Pentateuch; Genesis, Exodus, Leviticus, Numbers and Deuteronomy. The potential value of using such quantitative approaches is explored in relation to other forms of textual exegesis. Using a maximum likelihood approach, the degree distributions of the social networks are shown to approximate to a power law with exponential cutoff. The node representing Moses is very highly connected and falls outside the best fit line, as does the node representing Yahweh, which may indicate authorial emphasis. Only the social network from Genesis is assortative, a property typical of many real world social networks. A substantial proportion of disassortativity in the social network based around Moses disappears when the node is removed, potentially indicating some artificiality in its orientation within the network. The approximation of the degree distributions to a power law with exponential cutoff represents an emergent property resulting from the combinatorial and collaborative manner of composition, and indicates a bounding constraint on more highly connected nodes. Unusually highly connected nodes representing the deity and prophet may be characteristic of social networks derived from religious texts.

## Introduction 

Social network analysis represents individuals as nodes on a network, and their interactions as edges between those nodes, and is a fundamental approach to understanding social dynamics (Lazer et al. 2009). Statistical approaches have been important in characterizing the properties of real world complex networks, formed from large numbers of empirical observations, such as ecological, infrastructural, cellular, biochemical and social networks (Albert and Barabasi 2002). The past two decades have seen important insights into the properties of these networks and the characterization and identification of the universal properties of real world networks, including social networks, and the tools to characterize these statistical properties are widely incorporated into social network analysis. Some putative commonalities have been identified between these diverse networks. One of the most well-known is the ‘scale-free’ property. This refers to the relative frequency of nodes of different degrees (the number of edges of a node), which in real world networks have been proposed to approximate to a negative power law or ‘scale-free’ distribution (Barabasi and Albert 1999). While the property has been useful in the development of network growth models, the view of the ubiquity of scale-free distributions is being refined, with work showing that with more accurate fitting not all real world networks fit power laws with statistical certainty (Clauset et al. 2009), while others may follow alternative heavy tailed distributions such as a power law with exponential cutoff (D’Souza et al. 2007). Elucidation of other common and disparate features between networks, and their origins, is ongoing.

Recently, social network analysis approaches have begun to be used in the analysis of character relationships in texts, including mythological texts (Mac Carron and Kenna 2012; Mac Carron and Kenna 2013) and fiction (Waumans et al. 2015). Such networks can be termed ‘narrative networks’ and their study constitutes a nascent field. Statistical analysis of social interaction networks has rarely been utilized for the analysis of historical and religious texts. In particular, the Bible has been a center of western scholarly efforts for almost 2000 years, with a transition occurring over time from religious and philosophical interpretation, to a more scientific approach aimed at determining the authorship, mode of composition and historicity of the texts, and so may be amenable to statistically orientated social network approaches. The large amount of scholarly material concerning the Bible’s historicity, composition and interpretation is advantageous to understanding the statistical properties of networks derived from the text, and how they arose.

The foundation of the Jewish and Christian religions is based on the figure of Moses. The first five books of the Bible, the Pentateuch, give an account of the origin and development of the Jewish people and their religion through their early origin in the Levant, their subsequent time in Egypt, exit through the Sinai, and then initiation of the conquest of Transjordan. Moses is the central figure in this narrative, a character who guides his people from Egypt to a new homeland, whilst codifying religious ritual and a moral and legal framework. The actual historical figure of Moses has been a source of academic contention. On one extreme, he is viewed as a mostly fictional character formulated for political and social reasons (Noth 1981). On the other, by some scholars he has been viewed as a largely historical figure (Albright 1973). A lack of direct archeological evidence for the Israelite’s presence in Egypt, for their movement from station to station in the Sinai, and for their conquest of the cities of the Transjordan Levant (Finkelstein and Mazar 2007) has added to the debate. In *lieu* of direct material evidence, textual analysis of the Pentateuch has been a major source of effort. A battery of approaches have been employed, such as the parsing of textual variants (Anderson and GilesT 2012), source (Wellhausen 1883) and form (Noth 1981) criticism, identification of geographical locations (Oblath 2004), and the etymology of personal names (Hoffmeier 2005) (note, the references quoted are merely provided as exemplars of each approach, given the large volume of scholarly study dedicated to this topic), however a consensus on the mode of composition, which is connected to the degree of historicity contained in the Pentateuch, has not been achieved. Here, we utilize the novel approach of analyzing the statistical properties of the network formed by the interactions of characters from the Pentateuch to investigate the role of Moses in the text.

## Methods

For this study, the 2013 New World translation of the Bible was used. Individual characters represent nodes on the network. For consistency, the term ‘Yahweh’ is used for the deity throughout; the original Hebrew of the Pentateuch uses a combination of the tetragrammaton ‘YHWH’ and ‘Elohim’. Characters in Genesis starting with Abraham were included, as at this point the text begins to associate characters with discrete geographical locations, and so more clearly represent a potential historical narrative. Networks were constructed manually for Genesis, for the books of Exodus, Leviticus, Numbers and Deuteronomy (ELND), and for all books of the Pentateuch combined (Combined). The possible deity Azazel and angels are included as characters. In Exodus there are two Pharaohs, of the oppression and exodus, the first of which dies while Moses is in Midian (Exodus 2:23). A social connection is defined as collaboration, addressing directly or indirectly another character, family connection (parent-offspring and marriage, as with genealogical networks), inheritance, physical conflict or criticism. Kinship can be ambiguous between genders. Marriage and concubinage can be unequivocally described as kinship, however sexual relations are more ambiguous, here we classify them as kinship when consensual.

The size and density of a network may be inferred from *n*, the number of nodes, and *m*, the number of edges. The most common type of statistical analysis of networks is the degree distribution. This refers to the proportion of nodes *p(k)* that have a discrete number of connections, *k*. In empirical networks, *p*(*k)* has been proposed to often approximate to a negative power law distribution, termed ‘scale free’ (Barabasi and Albert 1999) as follows:

1 $$ p(k) \propto {k}^{-\gamma } $$

In order to test if the degree distribution follows a negative power law, maximum likelihood estimation (MLE) was used to fit the data to a log likelihood function describing the power law, as recommended by a number of authors (Clark et al. 1999; Goldstein et al. 2004; Newman 2005; Bauke 2007; White et al. 2008; Clauset et al. 2009). The probability density function (PDF) of a negative power law distribution is:

2 $$ p\left(K=k\right) = A{k}^{\mathit{\hbox{-}}\gamma } $$

where *A* is a normalization constant. The likelihood function is:

3 $$ \mathrm{\mathcal{L}}\left(\gamma; {k}_1,\dots, {k}_n\right)=A{\displaystyle {\prod}_{i=1}^n}{k}_i^{-\gamma } $$

*A* is equivalent to the following for discrete data (Newman 2005):

4 $$ A = \frac{1}{{\displaystyle {\sum}_{i=1}^{\infty }}{k}_i^{-\gamma }} $$

Consequently, the log likelihood *ℓ* may be expressed as:

5 $$ \ell \left(\gamma; {k}_1,\dots, {k}_n\right) = nln\left(\frac{1}{{\displaystyle {\sum}_{i=1}^{\infty }}{k}_i^{-\gamma }}\right)-\gamma {\sum}_{i=1}^n ln{k}_i $$

MLE was conducted on the degree distribution for each dataset, using Eq. (5), and utilizing the Broyden-Fletcher-Goldfarb-Shanno (BFGS) optimization procedure (Broyden 1970; Fletcher 1970; Goldfarb 1970; Shanno 1970). The same methodology was also followed for MLE of the log likelihood functions of the lognormal, Weibull and exponential distributions. Subsequently, a power law distribution with exponential cutoff was fitted to the degree distributions. This is a combination of a power law and exponential distribution, with the exponential component taking prominence in the right hand tail of the distribution, leading to a cutoff or decay from the straight line of the power law, when viewed on a log-log graph. The function is defined as:

6 $$ p\left(K=k\right) = B{k}^{-\gamma }{e}^{-\lambda k} $$

where *B* is a normalization constant. The data was fitted to the distribution by MLE utilizing the powerlaw R package (Clauset et al. 2009). Comparison of the fitted distributions was conducted by calculating the Akaike Information Criterion (AIC) (Akaike 1973) for each distribution, the lowest value of AIC indicating the best fitting distribution. A likelihood ratio test (LRT) was used to distinguish between the best models identified using AIC, when those models were nested, the degrees of freedom (df) being equal to the difference in free parameters between the two models.

The clustering coefficient, *C*, measures the tendency for nodes to cluster together, which reflects the probability with which two nodes connected to a common node are also connected with each other. *C*_*i*_, the clustering coefficient for node *i*, with *k*_*i*_ neighbors, and *m*_*i*_ edges between the neighbors of *i*, is calculated as follows (Watts and Strogatz 1998):

7 $$ {C}_i = \frac{2{m}_i}{k_i\ \left({k}_i - 1\right)} $$

The mean clustering coefficient of the entire network, $$ \overline{C} $$, is the average of all individual values of *C* for all the nodes of the network. $$ {\overline{C}}_{rand} $$ is the mean clustering coefficient of a random network. Here, we generate a random network using the values of *n* and *m* from the network with which the comparison is being made. We use the Erdos-Renyi *G(n,m)* model (Erdos and Renyi 1959) for generating the random graph. This assigns an equal probability to each node for the assignment of an edge, equivalent to $$ \raisebox{1ex}{$m$}\!\left/ \!\raisebox{-1ex}{$2n$}\right. $$. Often, clustering is greater in social networks in comparison to non-social networks, and this may be explained by the presence of communities within the network (Newman and Park 2003). The mean clustering coefficient per degree $$ \overline{C}(k) $$ is the mean clustering coefficient for all nodes in the network that possess a particular value of *k*.

When the average clustering coefficient per degree $\overline{C}(k)$ is plotted against *k*, decay indicates a hierarchical structure (Ravasz and Barabasi 2003; Dorogovtsev et al. 2001), by indicating that highly connected nodes have a lower clustering coefficient, characteristic of network hierarchicity. In hierarchical scale free networks, the average clustering coefficient per degree, $\overline{C}(k)$, shows the following relationship:

8 $$ \overline{C}(k) \propto {k}^{-\beta } $$

β takes the value of 1 in pseudofractal scale free networks (Ravasz and Barabasi 2003).

The average path length, $\overline{l}$, represents the average number of connections along the shortest paths for all possible pairs of nodes in a network. $\overline{l}$ is calculated as follows:

9 $$ \overline{l} = \frac{1}{n\left(n-1\right)}{\sum}_{i\ne j}l\left(i,\ j\right) $$

where *l(i,j)* is the shortest distance between nodes *i* and *j*. The concept of a small world network relates to the idea that a network may have a large value of $\overline{C}$, in comparison to the corresponding value from a random network, ${\overline{C}}_{rand}$, with the same values of *n* and *m*. In contrast, the respective average path lengths $\overline{l}$ and ${\overline{l}}_{rand}$ are roughly comparable (Watts and Strogatz 1998).

The assortativity of a network refers to the extent to which similarly connected nodes are connected to each other. The assortativity coefficient (*r*) represents the Pearson correlation coefficient of the degrees of the nodes at each end of an edge, *k* and *h*, and can be calculated as follows (Newman 2003):

10 $$ r=\frac{m^{-1\ }{\displaystyle {\sum}_i}{h}_i{k}_i-{\left[{m}^{-1}{\displaystyle {\sum}_i}\frac{1}{2}\left({h}_i+{k}_i\right)\right]}^2}{m^{-1}{\displaystyle {\sum}_i}\frac{1}{2}\left({h}_i^2+{k}_i^2\right)-{\left[{m}^{-1}{\displaystyle {\sum}_i}\frac{1}{2}\left({h}_i+{k}_i\right)\right]}^2} $$

If *r* is positive the network is considered assortative, while if negative then disassortative. *r* is usually positive in social networks, while negative in other types of network (Newman 2003), indicating interactions between similarly connected individuals in social networks, which seems to reflect the tendency of people to associate in groups of similar individuals (Newman and Park 2003; Newman 2003), a process termed ‘homophily’ (McPherson et al. 2001) (a review). This may also indicate the presence of substructures in the network. Different types of substructures can be observed in networks. Isolates are nodes unconnected to other nodes, pendants are nodes that have only one edge connected to another node, while triangles are comprised of three nodes connected with each other. The number of triangles in a network gives an indication of the degree of clustering, with a greater density of triangles indicating a greater level of clustering. In addition, triangles are particularly associated with social networks, due to a mechanism of triangle closing whereby links between X and Y, and X and Z, are expected to lead to formation of a social interaction between Y and Z (Granovetter 1973). Partitioning is a procedure used to divide a network into components. Typically, this is a computationally hard problem which necessitates the use of heuristics. Here, the method of Blondel et al. (Blondel et al. 2008) is utilized which uses modularity (Newman 2006) and an iterative approach to its improvement to achieve rapid partitioning.

The definition of a node as being peripheral or core utilizes the idea of a *k*-core (Seidman 1983), which refers to a subgraph of the network for which each node has a minimum degree, *k*. The *k*-coreness of each node refers to the maximally connected *k*-core to which it belongs. Betweenness centrality reflects the centrality of a node in a network and is quantified by the number of times a node is located along the shortest path between two other nodes (Freeman 1977). The betweenness centrality, ℂ_*B*_, of a node *z* may be represented as follows:

11 $$ {\mathrm{\mathbb{C}}}_B(z) = {\sum}_{i\ \ne j\ne z\in\ Z}\frac{\sigma_{ij}\kern0.5em (z)}{\sigma_{ij\kern0.5em }} $$

where *σ*_*ij*_ is the total number of shortest paths between nodes *i* and *j*, *σ*_*ij*_ (*z*) is the number of the shortest paths between node *i* and *j* that pass through node *z*, and *Z* is the set of nodes that comprise the graph. Finally, the closeness centrality, ℂ_*C*_, reflects the length of the average shortest path between a node and all remaining nodes in a graph (Sabidussi 1966), as follows:

12 $$ {\mathrm{\mathbb{C}}}_C(i)={\left[{\sum}_{j=1}^nl\left(i,j\right)\right]}^{-1} $$

where ℂ_*C*_(*i*) is the closeness centrality of node *i* and *l(i,j)* is shortest path between nodes *i* and *j*.

## Results and discussion

### Degree distributions

The degree distributions of the Genesis, ELND and Combined social networks were fitted to a variety of distributions, and AIC values generated for each fit (Table 1). In each case, the power law with exponential cutoff was the best model according to the AIC, with a power law being the next best model. The goodness of fit of the power law with exponential cutoff distribution was compared with that of the power law using a LRT with df = 1. The LRT determined that the power law with exponential cutoff model was significantly better in each case, with values of *p* = 2.97 × 10^-7^ for the Genesis network, *p* = 9.95 × 10^-8^ for the ELND network, and *p* = 2.09 × 10^-12^ for the Combined network.

**Table 1:** Statistics of MLE degree distribution analysis

PDF	Genesis network AIC values	ELND network AIC values	Combined network AIC values
Power law	1144.9	946.4	1915.2
Exponential	1293.9	1035.5	2134.9
Weibull	1291.0	1032.0	2125.6
Lognormal	1191.7	957.1	1957.5
Power law with exponential cutoff	1120.7	920.0	1863.9

**Fig. 1:**
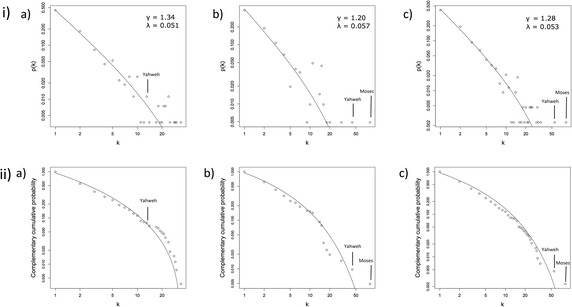
Degree distributions of the social networks of the Pentateuch. Degree distributions are shown for the social networks of (**a**) Genesis; **b** Exodus, Leviticus, Numbers and Deuteronomy (**c**) entire Pentateuch. Displayed are i) the PDF; ii) the CCDF. Nodes representing Moses and Yahweh are indicated. Best fit lines to a power law with exponential cutoff distribution were estimated as described in Methods, and the estimated values of γ and λ (Eq.(6)) are indicated on (i)

The degree distributions are displayed in Fig. 1, with a best fit line showing the estimated power law with exponential cutoff distribution for the PDFs, and the respective complementary cumulative probability distribution functions (CCDFs), where *p*(*K* ≥ *k*) is plotted versus *k*. For all three networks, the exponent for the power law component of the distribution were similar (*γ* = 1.34 for Genesis, *γ* = 1.20 for ELND and *γ* = 1.28 for Combined). Values of 2 ≤ *γ* ≤ 3 derived using MLE have been observed for social networks extracted from mythological texts when fitted to a power law (Mac Carron and Kenna 2012; Mac Carron and Kenna 2013). An exception is the degree distribution of the Iliad, which is better fitted by a power law with exponential cutoff, and here the exponent of the power law component is 1.51 (Mac Carron and Kenna 2012). The exponential decay has a similar scaling factor in each network (λ = 0.051 for Genesis, λ = 0.057 for ELND and λ = 0.053 for Combined). The exponential decay is better visualized on the CCDF plots in Fig. 1 (ii), leading to curvature in the right hand of the best fit line, while a simple power law is expected to produce a straight line on a log-log scale. Local structure has been shown to influence degree correlations in social networks (Newman and Park 2003), and this is likely to lead to differing values of *γ* between social networks derived from literature. Better understanding of the relationship between the local structure of the networks and their respective degree distribution may give some insights into how the narratives were formulated, as they may be characteristic of the mode of formulation.

Moses and Yahweh (with degrees of 82 and 55, respectively) are outliers to the best fit lines of the ELND and Combined networks (Fig. 1b and c). Such marked outliers are an unusual feature in social networks, and we have not been able to find a comparable example from the literature. Pertinently, we note that the central characters from Beowulf, the Táin Bo Cuailnge and the Iliad (Beowulf, Cú Chulainn and Achilles, respectively) do not fall outside the best fit lines of their respective degree distributions (Mac Carron and Kenna 2012) (these were initially fit using least squares regression and subsequently confirmed using MLE (Mac Carron 2014); personal communication Pádraig Mac Carron), and neither do those from the Icelandic Egil’s Saga (Egil) and Gisla’s Saga (Gisla) (Mac Carron and Kenna 2013). However, it should be noted that none of these best-fit lines, with the exception of the Iliad, display decay at higher degrees, unlike those reported here. Consequently, the elevated degrees of the Yahweh and Moses nodes within the narrative of the Pentateuch present a key difference. The node representing Yahweh is particularly highly connected in the Combined network and a partial explanation for this is that Yahweh is a transgenerational figure, spanning several generations across the entire narrative, and so should accumulate connections over generational time. Likewise, the relatively high degree of the Moses node in comparison to the Yahweh node will be reduced. This effect may be observed by comparing Fig. 1a, b and c.

**Fig. 2:**
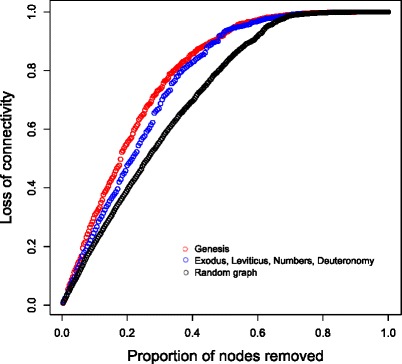
Attack tolerance of the networks. The Genesis (red) and ELND (blue) networks were subject to random node removal, and the subsequent effect on network connectivity (defined in (Matisziw et al. 2009), this refers to presence of a path between two nodes), using the NetSwan R package. For comparison, a random graph (black) based on the numbers of nodes and edges in the Genesis network was also examined

The Genesis and ELND networks show robustness to the removal of nodes, compared to a random network, with the Genesis network being more robust than the ELND network (Fig. 2). Such robustness is typical of heavy tailed distributions such as power laws (Albert et al. 2000). The removal of Moses from the ELND network results in a network more robust to node removal (data not shown), thus the presence of a highly connected node may affect the overall network properties.

### Substructure within the networks

**Fig. 3:**
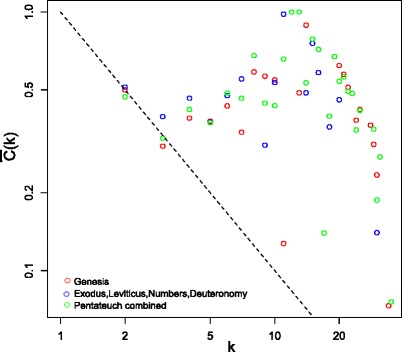
The variation in average clustering coefficient with node degree. The average clustering coefficient, $\overline{C}(k)$, was plotted against node degree, *k* for the three networks, indicated by different colors. The dotted line represents $\overline{C}(k) = {k}^{-1}$ ($\overline{C}(k) \propto {k}^{-1}$ is diagnostic of hierarchicity (Ravasz and Barabasi 2003))

**Fig. 4:**
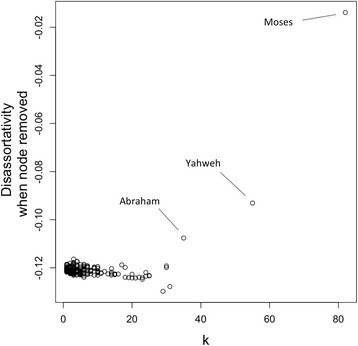
Nodes that affect disassortativity of the Combined network. The resulting disassortativity of the Combined network is shown, when each node is systematically removed versus the degree of the node. Indicated are the nodes that have the largest effect

Evidence of a relationship between the average clustering coefficient and node degree is difficult to discern for the three networks (Fig. 3). Such a relationship is indicative of hierarchicity in a network, and is commonly observed in social and other types of networks (Ravasz and Barabasi 2003). Assortativity is typical of social networks, but is not so commonly found in other types of networks (Newman 2003). In particular, the property appears to derive from correlating mechanisms deriving from the formation of social interactions, likely from a homophily mechanism whereby highly connected individuals have a preference to connect with other well connected individuals (Johnson et al. 2010). Only the Genesis network shows positive assortativity (*r* = 0.11, Table 2), while the ELND and Combined networks are disassortative (*r* = −0.23 and *r* = −0.12, respectively, Table 2). Interestingly, the removal of Moses from the ELND and Combined networks results in a reduction in disassortativity (*r* = −0.16 and *r* = −0.01, respectively), and it results in the biggest change in diassortativity when each node is systematically removed from the respective networks. This indicates that a proportion of the disassortativity in the two networks is due to this character. The next most influential node on disassortativity is Yahweh, with a resulting disassortativity of *r* = −0.20 and *r* = −0.09 for the ELND and Combined networks when the node is removed, respectively. The relationship between the resulting disassortativity value when a node is removed, and its node degree is shown in Fig. 4, for the Combined network. Only three nodes have an effect on disassortativity: those of Moses, Yahweh and Abraham. There are two potential explanations for why the three most highly connected nodes substantially impact disassortativity, while none of the other nodes do; either there is a degree threshold above which node removal may strongly impact assortativity or they are placed differently within the network.

**Table 2:** Properties of the social networks of the Pentateuch

Network	*n*	*m*	$\overline{k}$	$\overline{l}$	${\overline{l}}_{\mathrm{rand}}$	$\overline{C}$	γ	*λ*	*r*
Genesis	267	548	4.10	4.56	4.05	0.48	1.34	0.051	0.11
Exodus	92	200	4.35	3.42	3.16	0.48	0.98	0.092	−0.16
Numbers	135	283	4.19	3.16	3.57	0.34	1.29	0.055	−0.21
Exodus, Leviticus, Numbers and Deuteronomy (ELND)	205	469	4.58	3.26	3.58	0.53	1.20	0.057	−0.23
Pentateuch Combined	433	936	4.32	4.17	4.29	0.48	1.28	0.053	−0.12

The property of small worldness is due to clustering of nodes, characteristic of social networks. All three of the networks are small world; while the average path lengths of the networks are similar to those of the respective random graphs, the small world property is conferred by the higher degree of clustering in the three networks compared to random graphs (Table 2). The giant component, *Gc*, of a network refers to the largest component of a network, a component being a group of nodes that are all connected. Social networks typically have large giant components indicating that most of the members of the network are usually connected with each other. Here, the giant component represents 94 % of the Genesis network, 96 % of the ELND network and 94 % of the Combined network, indicating only a few isolates present in each network. Removal of the Moses node from the ELND and Combined networks results in a reduction in size of *G*_*c*_ to 92 % in each case. Interestingly, even though the Yahweh node has a lower degree than the Moses node, its removal results in a larger decrease in the relative size of the giant component: to 88 % and 91 % of the ELND and Combined networks, respectively. Given that centrality measures attempt to measure the importance of a node, these might be expected to reflect a higher centrality of the Yahweh node. However, in the Combined network *ℂ*_B_ = 2.32 × 10^4^ for the Yahweh node, while ℂ_*B*_ = 3.11 × 10^4^ for the Moses node and ℂ_*c*_ = 0.0364 for the Yahweh node and ℂ_*c*_ = 0.0365 for the Moses node. These measures do not show a greater centrality for the Yahweh node, and reflect the current lack of a measure that quantifies the influence of a node on *Gc* (Morone and Makse 2015). As a consequence, we utilized an empirical approach of systematically deleting each node individually and calculating the resulting decrease in size of *Gc* for each node. This allowed identification of the most important node on the size of *Gc*, which was the node corresponding to the character of Seir. This node is moderately connected with *k* = 8, the *k*-coreness is 2 which indicates a periphericity to the main narrative, while ℂ_*B*_ = 6.73 × 10^3^ and ℂ_*c*_ = 0.0336, which are both comparable to the respective values for Moses and Yahweh, however the node is influential structurally. Seir the Horite was the ancestor of the Horite chieftans Lotan, Shobal, Zibeon, Anah, Dishon, Ezer and according to Genesis gave his name to mountains of Seir, located in the Negev south of the Dead Sea. The original Horites were replaced by the Edomites and Seir’s offspring were figures in this transition within the narrative. The second most influential node on *Gc* was that of Yahweh, followed by Lot, and then Moses, indicating that simple node degree is not a predictor of impact on *Gc*.

**Fig. 5:**
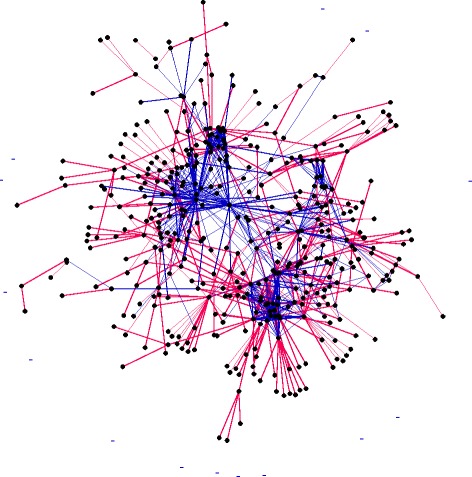
Social network of the Pentateuch. The giant component of the Combined social network of the Pentateuch is displayed. The network was drawn using Gephi (Bastian et al. 2009) utilizing the Hu algorithm (Hu 2006). Red edges are defined as kin interactions, while blue are other types of social interaction, some edges may be comprised of both types of interactions, in this case red supercedes blue

The three networks are mixed networks, comprised of social interactions such as conversation, verbal approbation or violence for example, and kin interactions. Consequently, it was decided to examine the kin interactions in more detail. Firstly, the proportion of edges in the network due to kin interactions is high: 49 % for the Genesis network, 32 % for ELND network, and 40 % for the Combined network (Table 3, although note that some edges that describe kin interactions also describe other social interactions). Likewise, the number of characters who are linked by kin in the form of a giant component is 72 % for Genesis, 34 % for the ELND network and 56 % for the Combined network (Table 3). This high proportion of kin interactions could be an artificial literary construction, or reflect that the population related in the narrative was actually very small. When represented on the network, the kin interactions can be seen as peripheral with a non-kin core (Fig. 5). This may be quantified using the *k*-coreness measure, with nodes connected by kin edges possessing an average *k*-core value of 3.45 (SD = 3.96), and nodes connected by non-kin edges possessing an average *k*-core value of 7.78 (SD = 4.08). These values are significantly different from each other (*p* < 0.0001, two tailed Student’s *t* test). Thus, to some extent the kin descriptions might be viewed as ornamental to the main narrative. This is consistent with the evidence that many of the genealogies were elaborated late in the development of the text (Johnson 2002).

**Table 3:** Additional characteristics of the social networks of the Pentateuch

Network	Number of kinship edges	Number of nodes that comprise the giant component of the kinship network	Number of pendants	Number of pendants in equivalent random graph	Number of triangles	Number of triangles in equivalent random graph	*G*_*c*_	Number of female characters	$\overline{C}$of female characters	$\overline{k}$ of female characters
Genesis	270	192	120	19	838	15	250	27	0.48	5.50
Exodus	87	58	35	3	259	11	85	8	0.44	3.38
Numbers	66	14	56	8	156	14	126	8	0.08	2.63
Exodus, Leviticus, Numbers and Deuteronomy (ELND)	148	69	79	11	746	13	197	15	0.31	4.40
Pentateuch Combined	371	244	178	30	1383	14	408	42	0.42	5.10

Network analysis allows the role of different categories of character to be examined and summarized. Interestingly, while female characters are in some senses minor in terms of the total number of characters, which is low (27 in the Genesis network, 15 in the ELND network and 42 in the Combined network, Table 3), they do not seem to have a markedly reduced average degree compared to the network as a whole ($\overline{k} = 5.50$ for the Genesis network, $\overline{k} = 4.40$ for the ELND network and $\overline{k} = 5.10$ for the Combined network, Table 3), and the average *k*-coreness for females nodes (3.16) is slightly higher than for male (2.72). Respective values for all characters are $\overline{k} = 4.10$ for the Genesis network, $\overline{k} = 4.58$ for the ELND network and $\overline{k} = 4.32$ for the Combined network (Table 2). These observations indicate that female characters play significant roles in the narrative, but are not recorded in such quantities as male characters. Many of the male characters are mentioned simply for their familial relationships, which may be regarded as a convention. This is reflected by the large number of pendants compared to equivalent random networks: pendants account for 22 % of edges in the Genesis network, 17 % of edges in the ELND network and 19 % of edges in the Combined network. For the equivalent random networks, pendants account for 3 % of edges in the Genesis network, 2 % of edges in the ELND network and 3 % of edges in the Combined network (Table 3). Many of the pendants result from kin interactions, and are thus located on the periphery of the network, as can be seen in Fig. 5. The networks also have a high proportion of triangles compared to random networks (838 versus 15 for the Genesis network, 746 versus 13 for the ELND network, 1383 versus 14 for the Combined network, Table 3). Triangles are one of the characteristics of social networks, and so in this regard the social networks of the Pentateuch are typical.

**Fig. 6:**
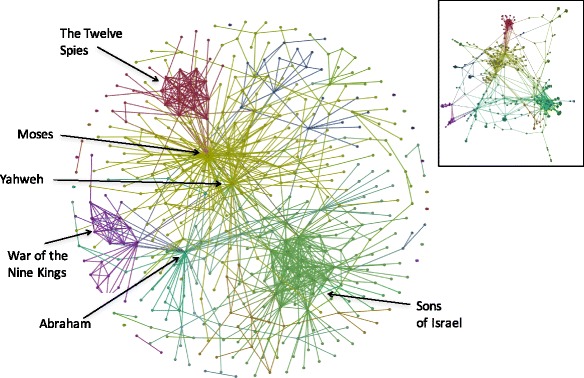
Partitioning of the Combined Pentateuch network. The Combined network was partitioned as described in Methods. The network was arranged according to the Fruchterman-Reingold algorithm (Fruchterman and Reingold 1991). Inset is a representation generated using the ForceAtlas2 algorithm (Jacomy et al. 2014). Different colors indicate the different major partitions identified

Partitioning was used to identify discrete communities of nodes within the network using modularity as the basis for partitioning (Fig. 6). The analysis reveals two major communities, centered respectively around the narratives regarding Moses in Exodus, Leviticus, Numbers and Deuteronomy, and the narrative regarding Jacob and the Sons of Israel in Genesis. Sub-narratives identified by the analysis include the War of the Nine Kings (Genesis 14), and the story of the 12 Spies (Numbers 13). Using an alternative representation, the network can clearly be seen to have three domains arranged as a triangle centered around Abraham, Jacob (the Sons of Israel) and Moses (Fig. 6, Inset). This structure reflects the three phases of the narrative, corresponding to three different generations, however generational phasing would not be expected to lead to partitioning of the graph in itself, as generations in human society are not discrete and sequential. Literary discontinuity between the narratives of Genesis and Exodus is central to the argument against an original core author, termed the ‘Yahwist’ (Schmid 2006), and is reflected in the partitioning of the Complete network. In addition, the partitioning is consistent with the idea that the narrative was comprised of major stand alone narrative units that were combined in a chronological fashion.

### Network topology and mode of composition

For comparative purposes, social network analyses of fictional social networks are limited. They include social network analyses of characters contained within the Marvel Universe (Alberich et al. 2002; Gleiser 2007), Victor Hugo’s Les Misérables, J.R.R. Tolkien’s Fellowship of the Rings, William Shakespeare’s Richard III and J.K. Rowling’s Harry Potter (Mac Carron and Kenna 2012), and the books of J.K. Rowling, G.R.R. Martin, Phillip Pullman, and Les Misérables (Waumans et al. 2015). Some of the respective degree distributions are reported to follow an exponential distribution (Mac Carron and Kenna 2012; Alberich et al. 2002), while some have been reported to follow a power law (Waumans et al. 2015; Gleiser 2007). In addition, there is a tendency towards disassortativity (Mac Carron and Kenna 2012). However, presently there are insufficient analyses in the literature to clarify if there are key differences with real world social networks.

The Pentateuch is considered literary and ahistorical by some scholars, representing the ‘minimalist’ viewpoint (Thompson 1987; Lemche 2008), while at the opposite extreme, the text is viewed as being a significant element of accuracy (Hoffmeier 1999; Kitchen 2003). Perhaps the most common scholarly view is somewhere between these two positions (Finkelstein and Mazar 2007; Dever 2003). Intimately connected with historicity, the authorship of the Pentateuch has been much debated, centering on the number of authors and date of composition. There is a consensus that there was more than one author, and that editing and revision of the text occurred. Given a tentative date of the middle to late 13^th^ century BCE for the passage through the Sinai (Dever 2003), and that writing was present in the Levant and surrounding regions at that time, then it would seem possible at least that a historical record survived in written form. However, it would appear that the first major component of the text was written a considerable period of time after this date, discussed further below. Thus, while the social network may potentially possess an historical imprint in its structure, it is difficult to validate from internal considerations.

That such a high proportion of characters in the ELND network are related to each other is remarkable, and likely represents a priestly or monarchical pressure to record and emphasize ancestral kin within the narrative (Johnson 2002). Genealogical lists are a feature of many ancient texts such as the Iliad, and the network approach allows a better quantification of this component of the text. The large proportion of pendants (19 % of all edges for the Combined network; Table 3) indicates an emphasis on lists in the narrative, and the percentage kin component is also high for all three networks, as discussed. For comparative purposes, the percentage kin component of different narratives may be calculated and this may help in classifying different forms of narrative.

The figure of Moses has been viewed as at least partly mythic, suggested by various lines of textual evidence (Noth 1981), and the uncertainty is exacerbated by the lack of archeological evidence for the existence of Israelites at the proposed time of the Exodus, or contemporaneous references to him outside the Bible. In our analysis, he is part of a network with some similarities to real world social networks. The interpretation of the highly connected nature of the node representing Moses are various. Firstly, the biblical Moses may represent an amalgamation of characters retrojected onto a historical stratum (Noth 1981), the amalgamation process leading to the unusually high degree. Here, comparison with the network characteristics of composite characters from other texts would be helpful. Secondly, exceptionally strong emphasis may have been placed on the character during the composition of the core narrative. Such an original core narrative has been attributed to an author termed the ‘Yahwist’, however his or her existence is contentious (Dozeman and Schmid 2006). The so-called Yahwist’s contribution to the Pentateuch would appear to be no earlier than the 8^th^ Century BCE (Van Seters 1975), which is distant from the time of occurrence of the events described in the narrative. Such a mechanism of composition may have given Moses a higher relative degree than is typical for the main characters in fictional narratives, and can be explained by the religious emphasis placed on him, resulting in an elevation of his role within the text. More prosaically, the ELND network may possess characteristics and motifs of a biography, however currently there is a lack of social network analyses of such texts.

In our opinion, understanding the mechanistic origin of the highly connected nature of the Moses node during composition, will involve comprehensive comparison with social networks derived from historical, religious, mythological, fictional, biographical and composite texts, and elucidation of their characteristic social network motifs. In addition, other characters may have been relatively exaggerated or minimized as the text of the Pentateuch developed. For example, the argument has been made that the characters of Aaron, Miriam, Abraham and Jacob have been relatively emphasized, while those such as Isaac, Nadab and Abihu have been minimized (Noth 1981). Reasons for the relative emphasis and de-emphasis of characters have been attributed to the political and social environment at the time of narrative development (Noth 1981). Given these potential diverse dynamics during composition, the observed approximation to a power law with exponential cutoff of the Combined network degree distribution is significant as it represents an emergent property of the compositional process more complex than one derived from a simple preferential attachment model.

The presence of assortativity in the Genesis network is a typical feature of real world social networks. Assortativity in real world social networks results from the phenomenon of homophily, which is a process of ‘like’ associating with ‘like’, and assigns factors such as ethnicity, religion and age as determinants of social contact (McPherson et al. 2001). Thus, its appearance in the Genesis network is curious, given that the Pentateuch is likely a composite of different sources and not the work of a single author. The homophilic mechanism operating therefore may not have been determined by social interactions, but could be a result of the process of composition, and the manner in which characters were linked with each other by the compositors, only a proportion of which may be historically accurate. In contrast, the ELND network shows disassortativity, a large portion of which is due to Moses, and so the probable authorial bias that elevated his role in the narrative might be observed via its effect on overall network properties.

The mechanism of composition of the Pentateuch is unsettled. For many years, the Documentary hypothesis prevailed; this proposed that the text was a combination of four independent documents (Wellhausen 1883), however this view has now dissipated into a series of different scenarios. Foremost amongst these are the Supplementary hypothesis, which proposes ‘a successive supplementation of one source or author by another’ (Van Seters 1975), and the Fragmentary hypothesis that proposes that a mass of written fragments were assembled by a single author (exemplified by (Whybray 1987)). The mechanism of network growth that gave rise to the power law with exponential cutoff distributions might shed light on this topic. The production of simple scale free degree distributions in real world networks is usually attributed to a mechanism of preferential attachment (Barabasi and Albert 1999), also termed the ‘Matthew effect’ (Merton 1968), a ‘rich get richer’ model whereby the probability of a node acquiring a new edge during network growth is proportional to the node degree. However, the model in its simplest form is not an adequate explanation for the distributions described here, which are better fitted by the more complex power law with exponential cutoff distribution. Degree distributions that display a power law with exponential cutoff have been observed in such real world social networks as scientific (Newman 2001a) and mathematical collaboration networks (Grossman 2002; Fenner et al. 2007), and also in the social network derived from the Iliad (Mac Carron and Kenna 2012). Models that incorporate more complex scenarios such as edge removal might be expected to more accurately model the manner in which the social networks of the Pentateuch evolved.

The manner in which heavy tailed distributions arise in social networks is unclear. The preferential attachment model appears consistent with how some types of social networks grow (Newman 2001b), while a more sophisticated version that incorporates anti-preferential attachment is able to produce assortativity, which the basic preferential attachment model is unable to do (Sendian-Nadal et al. 2016). Several modifications to the preferential attachment model have been proposed, that produce degree distributions approximating to power laws with exponential cutoff (Amaral et al. 2000; Dorogovtsev and Mendes 2000; Mossa et al. 2002; D'Souza et al. 2007). These models involve adding constraints to highly connected nodes that inhibit edge addition, proportional to their degree. The exponential decay observed in some real world social networks reflects a constraint on the upper number of an individual’s interactions, potential constraints could include limits to social cognition (Dunbar 2012). However, during the creation of social networks within narratives it is likely that mechanisms that produce heavy tailed distributions differ from those in real world social networks. For example, while during the composition of a narrative a process similar to preferential attachment might be followed in the development of characters and the formation of links with other characters within a narrative, the formation of interactions are not due to the same factors as observed in real world networks if there is a fictional component. Therefore, while the exponential decay represents the presence of a bounding factor on the number of interactions ascribed to more highly connected characters in the text, this is not necessarily due to the same mechanism(s) as in real world social networks. The presence of bounding on the number of edges makes the more highly connected nature of the Yahweh and Moses nodes more unusual.

**Fig. 7:**
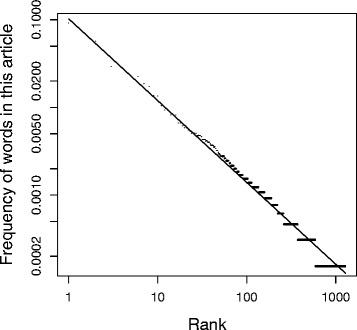
Popularity rank of words in this article versus frequency: a local illustration of Zipf’s Law. All words from this article except from the References list, Tables and the Figures were extracted, and their popularity rank was plotted against frequency. The power law best fit line was parameterized using MLE, as described in Methods; γ = 0.93

The matter takes on particular importance when considering the social networks of the Pentateuch, given that the mechanism by which the degree distributions arose may give some insight into the mode of composition. *Vice versa*, better understanding the mechanism producing the observed patterns may inform the formation of degree distributions in other networks consistent with a power law with exponential cutoff. What appears clear is that the property is emergent from the process of narrative composition and assembly, and that multiple authors may have unconsciously collaborated in the process. This reflects a process of self-organization whereby microscopic interactions are able to produce macroscopic order. In the same way that artists appear to reproduce fractal patterns because they are pleasing to the eye, being a reflection of physical forms commonly found in nature (Aks and Sprott 1996; Spehar et al. 2003; Redies et al. 2007), the compositors of the Pentateuch may have arranged the text so that the narrative and consequently the social network was aesthetically pleasing, religiously inspiring, or ‘realistic’, thus subconsciously promoting the form of their degree distributions (Czachesz 2012). Simple assembly of preexisting fragments into a whole would imply no such input, however such an assembly process would still require that all the components were modified so they formed a contiguous and consistent narrative, and this may have facilitated the emergence of the scale free distribution. By way of analogy, this article itself follows Zipf’s law (Fig. 7), which predicts that the popularity rank of words are related to their frequency by a power law distribution with exponent -1 (Zipf 1936). This is an emergent property of uncertain cause, but given that this article was composed from an amalgamation of disparate sources and themes, it provides a parallel to the emergence of the degree distributions during the composition of the Pentateuch. Whether the relative degrees of the characters in the text emerged from localized instances of authorial intent or passively from simple assembly mirrors the emergent beneficial traits of biological systems, most of which have arisen by the direct action of natural selection, but some of which may have arisen neutrally (Massey 2015).

## Conclusion

We have shown that analysis of the social networks of the Old Testament might be a useful complement to textual and archaeological considerations. Analysis of social networks derived from textual narratives potentially allows the identification of characteristic network motifs, and holds promise for the summation, comparison and analysis of diverse narratives. We show that while the narrative of the Pentateuch shows a number of properties characteristic of real world social networks, there are also a number of unusual features. Foremost amongst these are the highly connected nature of the nodes representing Moses and Yahweh in relation to the best fit lines to the degree distribution, and we propose that such emphasis on the prophet and deity might be a characteristic feature of religious texts as opposed to purely fictional texts; it is an open question as to whether a similar property is observed in social networks found in autobiographies and biographies, given their focus on a single individual. In addition, it is likely that the power law with exponential cutoff degree distributions have resulted from the process of composition; it remains to be determined if the parameter values of the fitted distributions, and the relative levels of assortativity and hierarchicity, can reveal further insights into the nature and mechanism of composition.

## References

[CR1] Akaike H, 1973 Information theory and and extension of the maximum likelihood principle. [ed.] Caski BN, Petrov F. Akademiai Klado. Proceedings of the second international symposium on information theory. s.l. pp. 267-281

[CR2] Aks DJ, Sprott JC (1996). Quantifying aesthetic preference for chaotic patterns. Empir Stud Arts.

[CR3] Alberich R, Miro-Julia J, Rossello F, 2002 Marvel universe looks almost like a real social network. arXiv:cond-mat/0202174.

[CR4] Albert R, Barabasi A-L (2002). Statistical mechanics of complex networks. Rev Mod Phys.

[CR5] Albert R, Jeong H, Barabasi A-L (2000). Error and attack tolerance of complex networks. Nature.

[CR6] Albright WF (1973). From the Patriarchs to Moses II. Moses out of Egypt. Biblical Archaeologist.

[CR7] Amaral LAN, Scala A, Barthelemy M, Stanley HE (2000). Classes of small-world networks. Proc Natl Acad Sci U S A.

[CR8] Anderson RT, Giles T, 2012 The Samaritan Pentateuch. An introduction to its origin, history, and sigfificance for biblical studies. [ed.] Tom Thatcher. Atlanta, Society of Biblical Literature

[CR9] Barabasi AL, Albert R (1999). Emergence of scaling in random networks. Science.

[CR10] Bastian M, Heymann S, Jacomy M (2009). Gephi: an open source software for exploring and manipulating networks.

[CR11] Bauke H (2007). Parameter estimation for power-law distributions by maximum likelihood methods. Eur Phys J B.

[CR12] Blondel VD, Guillaume J-L, Lambiotte R, Lefebvre E (2008). Fast unfolding of communities in large networks. J Stat Mech.

[CR13] Broyden CG (1970). The convergence of a class of double-rank minimization algorithms. J Inst Math Appl.

[CR14] Clark RM, Cox SJD, Laslett GM (1999). Generalizations of power-law distributions applicable to sampled fault-trace lengths: model choice, parameter estimation and caveats. Geophys J Int.

[CR15] Clauset A, Shalizi CR, Newman MEJ (2009). Power-law distributions in empirical data. SIAM Rev.

[CR16] Czachesz I (2012). God in the fractals: recursiveness as a key to relgious behaviour. Method Theory Study Relig.

[CR17] Dever WG (2003). Who were the early Isrealites and where did they come from?.

[CR18] Dorogovtsev SN, Mendes JFF (2000). Evolution of networks with aging of sites. Phys Rev E.

[CR19] Dorogovtsev SN, Goltsev AV, Mendes JFF, 2001 Pseudofractal scale-free web. arXiv:cond-mat/0112143v1 10.1103/PhysRevE.65.066122 12188798

[CR20] Dozeman TB, Schmid K (2006). A farewell to the Yahwist?.

[CR21] D’Souza RM, Borgs C, Chayes JT, Berger N, Kleinberg RD (2007). Emergence of tempered preferential attachment from optimization. Proc Natl Acad Sci U S A.

[CR22] Dunbar RIM (2012). Social cognition on the internet: testing constraints on social network size. Phil Trans R Soc B.

[CR23] Erdos P, Renyi A (1959). On random graphs. Publicaciones Mathematicae.

[CR24] Fenner T, Levene M, Loizou G (2007). A model for collaboration networks giving rise to a power-law distribution with an exponential cutoff. Soc Netw.

[CR25] Finkelstein I, Mazar A, 2007 The Quest for the Historical Israel. [ed.] B.B. Schmidt. Atlanta, Society of Biblical Literature

[CR26] Fletcher R (1970). A new approach to variable metric algorithms. Comput J.

[CR27] Freeman L (1977). A set of measures of centrality based on betweenness. Sociometry.

[CR28] Fruchterman TMJ, Reingold EM (1991). Graph drawing by force-directed placement. Softw Pract Exp.

[CR29] Gleiser PM, 2007 How to become a superhero. J Statistical Mechanics: Theory and Experiment, p. P09020

[CR30] Goldfarb D (1970). A family of variable metric updates derived by variational means. Math Comput.

[CR31] Goldstein ML, Morris SA, Yen GG (2004). Problems with fitting to the power-law distribution. Eur Phys J B.

[CR32] Granovetter MS (1973). The strength of weak ties. Am J Sociol.

[CR33] Grossman J (2002). Patterns of collaboration in mathetmatical research. SIAM News.

[CR34] Hoffmeier JK (1999). Israel in Egypt: the evidence for the authenticity of the Exodus tradition.

[CR35] Hoffmeier JK (2005). Egyptian personal names and other Egyptian elements in the Exodus-Wilderness narratives.

[CR36] Hu Y (2006). Efficient, high-quality force-directed graph drawing. Math J.

[CR37] Jacomy M, Venturini T, Heymann S, Bastian M (2014). ForceAtlas2, a continuous graph layout algorithm for handy network visualization designed for the Gephi software. PLoS One.

[CR38] Johnson MD (2002) The purpose of the Biblical genealogies, with special reference to the setting of the genealogies of Jesus. Eugene : Wipf and Stock.

[CR39] Johnson S, Torres JJ, Marro J, Munoz MA (2010). The entropic origin of disassortativity in complex networks. Phys Rev Lett.

[CR40] Kitchen KA (2003). On the reliabiity of the old testament.

[CR41] Lazer D (2009). Computational social science. Science.

[CR42] Lemche NP (2008). The Old testament between theology and history.

[CR43] Mac Carron P, Kenna R (2012). Universal properties of mythological networks. Europhysics Lett.

[CR44] Mac Carron P, Kenna R (2013). Network analysis of the Islending sogur - the sagas of the Icelanders. Eur Phys J B.

[CR45] Mac Carron P, PhD, 2014 Thesis: A network theoretic approach to comparative mythology. [ed.] Supervisor: Ralph Kenna. Applied Mathematics Research Center, Coventry University

[CR46] Massey SE (2015). Genetic code evolution reveals the neutral emergence of mutational robustness and information as an evolutionary constraint. Life.

[CR47] Matisziw TC, Murray AT, Grubesic TH (2009). Exploring the vulnerability of network infrastructure to disruption. Ann Reg Sci.

[CR48] McPherson M, Smith-Lovin L, Cook JM (2001). Birds of a feather: homophily in social networks. Annu Rev Sociol.

[CR49] Merton RK (1968). The Matthew effect in science. Science.

[CR50] Morone F, Makse HA (2015). Influence maximization in complex networks through optimal percolation. Nature.

[CR51] Mossa S, Barthelemy M, Stanley HE, Amaral LAN (2002). Truncation of power law behavior in “scale-free” network models due to information filtering. Phys Rev Lett.

[CR52] Newman MEJ (2001). The structure of scientific collaboration networks. Proc Natl Acad Sci U S A.

[CR53] Newman MEJ (2001). Clustering and preferential attachment in growing networks. Phys Rev E.

[CR54] Newman MEJ (2003). Assortative mixing in networks. Phys Rev Lett.

[CR55] Newman MEJ (2005). Power laws. Pareto distributions and Zipf’s law. Contemp Phys.

[CR56] Newman MEJ (2006). Modularity and community structure in networks. Proc Natl Acad Sci U S A.

[CR57] Newman MEJ, Park J (2003). Why social networks are different from other types of networks. Phys Rev E.

[CR58] Noth M, (1981) A history of Pentateuchal traditions. [trans.] Bernard W. Anderson. Chico, Scholars Press

[CR59] Oblath MD (2004). The Exodus itinerary sites.

[CR60] Ravasz E, Barabasi AL (2003). Hierarchical organization in complex networks. Phys Rev E.

[CR61] Redies C, Hasenstein J, Denzler J (2007). Fractal-like image statistics in visual art: similarity to natural scenes. Spat Vis.

[CR62] Sabidussi G (1966). The centrality index of a graph. Psychometrika.

[CR63] Schmid K, 2006 The so-called Yahwist and the literary gap between Genesis and Exodus. [ed.] Schmid TB, Dozeman K. A farewell to the Yahwist? Atlanta: Society of Biblical Literature

[CR64] Seidman SB (1983). Network structure and minimum degree. Soc Netw.

[CR65] Sendian-Nadal I, Danziger MM, Wang Z, Havlin S, Boccaletti S (2016). Assortativity and leadership emerge from anti-prefential attachment in heterogenous networks. Nat Sci Rep.

[CR66] Shanno DF (1970). Conditioning of quasi-Newton methods for function minimization. Math Comput.

[CR67] Spehar B, Clifford CWG, Newell BR, Taylor RP (2003). Universal aesthetic of fractals. Comput Graph.

[CR68] Thompson TL (1987). The origin tradition of ancient Israel.

[CR69] Van Seters J (1975). Abraham in history and tradition.

[CR70] Watts DJ, Strogatz SH (1998). Collective dynamics of ‘small-world’ networks. Nature.

[CR71] Waumans MC, Nicodeme T, Bersini H (2015). Topology analysis of soical networks extracted from literature. PLoS One.

[CR72] Wellhausen J (1883). Prolegomena to the history of Israel.

[CR73] White EP, Enquist BJ, Green JL (2008). On estimating the exponent of power-law frequency distriibutions. Ecology.

[CR74] Whybray RN 1987 The making of the Pentateuch. Sheffield: Journal for the Study of the Old Testament, 1987

[CR75] Zipf GK (1936). The psycho-biology of language.

